# *Saksenaea dorisiae* sp. nov., a New Opportunistic Pathogenic Fungus from Europe

**DOI:** 10.1155/2019/6253829

**Published:** 2019-09-22

**Authors:** Roman Labuda, Andreas Bernreiter, Doris Hochenauer, Christoph Schüller, Alena Kubátová, Joseph Strauss, Martin Wagner

**Affiliations:** ^1^Department for Farm Animals and Veterinary Public Health, Institute of Milk Hygiene, Milk Technology and Food Science, University of Veterinary Medicine Vienna, Veterinärplatz 1, 1210 Vienna, Austria; ^2^Research Platform Bioactive Microbial Metabolites (BiMM), Konrad Lorenz Strasse 24, 3430 Tulln a.d. Donau, Austria; ^3^Department of Applied Genetics and Cell Biology, Fungal Genetics and Genomics Laboratory, University of Natural Resources and Life Sciences, Vienna (BOKU), Konrad Lorenz Strasse 24, 3430 Tulln a.d. Donau, Austria; ^4^Charles University, Faculty of Science, Department of Botany, Culture Collection of Fungi (CCF), Benátská 2, 128 01 Prague 2, Czech Republic

## Abstract

A new species, *Saksenaea dorisiae* (Mucoromycotina, Mucorales), isolated from a water sample originating from a private well in Manastirica, Petrovac, in the Republic of Serbia (Europe), is described and illustrated. The new taxon is well supported by multilocus phylogenetic analysis that included the internal transcribed spacer (ITS) region, domains D1 and D2 of the 28S rRNA gene (LSU), and translation elongation factor-1*α* gene (tef-1*α*), and it is resolved in a clade with *S. oblongispora* and *S. trapezispora*. This fungus is characterized by its moderately slow growth at 15 and 37°C, sparse rhizoids, conical-shaped sporangia, and short-cylindrical sporangiospores. *Saksenaea dorisiae* is a member of the opportunistic pathogenic genus often involved in severe human and animal mucormycoses encountered in tropical and subtropical regions. Despite its sensitivity to several conventional antifungals (terbinafine and ciclopirox), the fungus can potentially evoke clinically challenging infections. This is the first novel taxon of the genus *Saksenaea* described from the moderately continental climate area of Europe.

## 1. Introduction

The genus *Saksenaea* S. B. Saksena is a mucoralean microscopic fungus (Mucoromycotina, Mucorales, Saksenaeaceae) comprising of species often causing severe human and animal cutaneous mucormycoses in both immunocompromised and immunocompetent hosts [1, 2]. The genus was first described from a forest soil in India in 1953. *Saksenaea vasiformis* S. B. Saksena is the only species of the genus (nowadays considered as *S. vasiformis* species complex) for more than 50 years reported in soil, drift-wood, and grains [1]. Interruption of skin integrity, such as needle sticks, insect stings or spider bites, and burn and accident wounds (up to 95% of cases), represents the most common mode of infection by this fungus from contaminated soil or water [2–4]. In addition to these traumatic implantations, infections through inhalation of spores and the use of indwelling catheters have been reported [5]. Species in this genus are responsible for skin infections and are characterized by rapid progression and invasion of neighboring tissues. Infection of the *Saksenaea* spp. is diagnosed by angioinvasion leading to tissue necrosis with cutaneous and subcutaneous involvement. Furthermore, rhino-orbito-cerebral and disseminated infections have been reported [1, 2]. Classic management of the infection site usually includes a combination of broad, aggressive and repeated surgical debridement (which may lead to amputation), and long-term systemic therapy with appropriate antifungals, preferably liposomal amphotericin B and/or posaconazole [6–9]. Antifungal therapy alone seems to be inadequate to control infection [2].

Recent revisions by Alvarez et al. [1] and Crous et al. [10, 11] applied a polyphasic approach, which includes multilocus (ITS, LSU, and tef-1*α*), revealed that the monotypic genus *Saksenaea* is genetically heterogeneous and encompassed more species, namely, *S. erythrospora*, *S. loutrophoriformis*, *S. oblongispora*, *S. trapezispora*, and *S. vasiformis* complexes (with more putative cryptic species). So far, these species (except *S. oblongispora*) have been reported worldwide from human and animal clinical cases. They were encountered in tropical and subtropical climates and have been reported from Africa (Tunisia), Australia, India, the Middle East (Iraq, Israel, and Saudi Arabia), New Zealand, Sri Lanka, South America (Colombia, Ecuador, and French Guiana), Thailand, and the USA [1, 12, 13]. In Europe, there are only a few published cases available reporting *S. vasiformis* infections, e.g., from Spain [3, 14–16], France [1, 13], and Greece [2]. Up to now, approximately 45 cases of mostly cutaneous infections of *Saksenaea*, have been reported worldwide [2, 4, 5, 10, 11, 13], although the actual number of clinical cases is possibly underestimated [4]. Despite its association in severe human infections, this genus remains a poorly studied mucoralean genus, mainly due to the lack of sporulation on the mycological culture media (e.g., Sabouraud dextrose agar, malt extract agar, and corn meal agar) routinely used in clinical laboratories [1].

During our microbiological survey of water samples, a fast-growing nonsporulating mucoralean fungus was recovered on a *Pseudomonas* selective agar plate in a sample originating from a private well in a rural area of Manastirica (the Republic of Serbia) in October 2018. This isolate was designated BiMM-F232 and further characterized in terms of morphology, physiology, molecular phylogeny, and antifungal susceptibility. Phylogenetically informative sequences were obtained from three loci, i.e., internal transcribed spacer region including 5.8S rDNA (ITS), domains D1 and D2 of the 28S rRNA gene (LSU), and the translation elongation factor-1*α* locus (tef-1*α*). Overall, the resulting data revealed that this isolate represents a novel species of the opportunistic pathogenic genus *Saksenaea*, and it is described and illustrated here as *Saksenaea dorisiae* sp. nov.

## 2. Materials and Methods

### 2.1. Sample Collection and Isolation of the Fungus

A single sample of water from a private well in Manastirica, Petrovac (the Republic of Serbia, Europe) was collected in October 2018. A 100 mL aliquot was aseptically filtered through a filter paper disc (0.45 *μ*m, 47 mm diameter, GN-6 Metricel, Pall, Mexico) plated on *Pseudomonas* selective agar amended with 1% v/v glycerol (Carl Roth, Germany) and incubated for 3 days at 37°C (±0.2°C) in the dark.

### 2.2. Cultivation of a Strain, Media, and Morphological Analysis

For phenotypic characterization, the strain was transferred (one agar block grown on CYA, ca 5 × 5 mm, in the middle) on potato dextrose agar (PDA, Fluka), malt extract agar (MEA, Merck), corn meal agar (CMA, Oxoid), Sabouraud 4% dextrose agar (SDA, VWR), water agar 1% (WA), oatmeal agar (OA), synthetic nutrient-poor agar (SNA), Czapek's agar (CZA), Czapek yeast extract agar (CYA), and yeast extract sucrose (YES) agar as described by Samson et al. [17] and incubated for 5–30 days in the dark at 25°C. Colony size (in mm), colony structure, and characteristics were noted after four days. However, the cultivation was prolonged up to 3 months on all media in order to observe and record changes in pigmentation of the colonies, as well as to determine the onset of sporangia and zygospore formation. For sporangial development, a recommended method of floating agar blocks in yeast extract water according to Padhye and Ajello [18] was used in this study. In order to determine the optimal and minimal/maximal temperatures for growth, the strain was incubated on four different media (CZA, CYA, PDA, and MEA) at 12, 15, 20, 25, 30, 35, 37, 39, 40, and 42°C (±0.1-0.2°C). Colony diameters were measured on the 4^th^ day of cultivation. For comparative description of the macroscopic and microscopic characteristics, CZA was used according to Alvarez et al. [1] and Crous et al. [10, 11].

The capability of three different vegetative forms of the fungus to germinate or grow on more extreme temperatures was assessed by incubation at 5 ± 0.3°C during 1 to 50 days or at 40 ± 0.1°C for 24 to 48 hours. For these tests the following procedure was applied: (a) spores: 100 *μ*L of spore suspension (4.0 × 10^5^ CFU/mL physiologic solution) collected from CZA (6 days at 30°C) was applied on MEA plate (at 37°C) at defined time intervals (1, 2, 3, 4, 7, 14, 21, 28, 42, and 50 d); (b) mycelial pellets: microcolonies of ca 50–200 *μ*m in diameter (∼2.0 × 10^4^/mL) after 10–12 d incubation submerse YES 5%, 140 rpm, 25°C, 100 *μ*L of pellet suspension applied on MEA plate (at 37°C); (c) mycelium: as a nonsporulating colony growing on MEA plate after 4 d incubation (37°C). Both spores and pellets were exposed to 5°C in physiological or YES broth liquids, respectively, and then transferred (100 *μ*L) onto a plate at 37°C. For exposure to 40°C, they were directly applied onto plates and after 24 and 48 hours transferred on a new MEA plate and further incubated at 37°C. Germination of spores was determined *in situ* under low magnification (50–100x).

A dried herbarium specimen of the holotype was deposited in the herbarium of the Mycological Department, National Museum, Prague, Czech Republic (PRM); the ex-type cultures were deposited in the Bioactive Microbial Metabolites (BiMM), Fungal Collection, UFT-Tulln (AT), and in the Culture Collection of Fungi (CCF), Prague (CZ).

For determination of microscopic traits, CZA was used after 6–14 days. Sporangiophore structures and sporangia formation were observed *in situ* under low magnification (50–100x). Details of sporangiophores, sporangia, sporangiospores, and other microscopic structures, such as width of hyphae, were observed in mounts with lactophenol blue (RAL Diagnostics). Sporangiospores were measured in water to prevent their deformation. For hyphae width, 25 measurements were done and represented as maximal value in *μ*m. For sporangiophore length and width and sporangial length (including columella and neck), 60 measurements were done, and they were represented as (minimal-), typical range and (-maximal) value in *μ*m. For sporangial venter, including columella (width), a range of mostly measured values with a maximal value of the venter width was represented. For sporangial neck (length and width), the measurements were represented as a range of minimal and maximal values. For sporangiospores, 70 measurements were done, and they were represented as (minimal-), typical range and (-maximal) value, including a mean and standard deviation.

Except on CZA, formation of sporangia was not observed, in any of the other media used after 8 days of cultivation. For these media incubation was prolonged for 3 months and the plates were checked at 5-day intervals for the onset of sporangia production. The photomicrographs were taken using a Motic BA310 microscope with Motic Image Plus 3.0 software. Lactophenol blue was used as a mounting medium for microphotography. Final microscopic pictures were black and white inverted. Photographs of the colonies were taken with a Sony DSC-RX100.

### 2.3. Antifungal Susceptibility Testing

A 50 *μ*L aliquot of sporangiospore suspension (4.0 × 10^5^ CFU/mL) of the *Saksenaea* strain BiMM-F232 (collected from CZA grown at 30°C for 14 days into physiological solution) was evenly distributed on RPMI-1640 (Sigma) medium plates. In addition to the spores, a 50 *μ*L aliquot of suspension of mycelial pellets (microcolonies sized roughly 50–200 *μ*m in diameter collected from YES 5% liquid medium grown at 25°C, 150 rpm after 10 days) were inoculated at a density of 2.0 × 10^4^/mL in the same way on the RPMI plates. Antifungal susceptibility testing was performed by applying the antifungal discs with 10 and 15 *μ*g amphotericin B, 10 *μ*g griseofulvin, 5 *μ*g caspofungin, 1 *μ*g flucytosine, 100 IU nystatin, 10 *μ*g econazole, 25 and 100 *μ*g fluconazole, 1 *μ*g voriconazole, 10 and 15 *μ*g ketoconazole, 5 *μ*g posaconazole, 10 *μ*g miconazole, 8 and 50 *μ*g itraconazole, and 50 *μ*g clotrimazole (Antifungal Discs, Liofilchem Diagnostici, Via Scozia, Italy) and 50 *μ*g ciclopirox and 30 *μ*g terbinafine (Neo-Sensitabs, Rosco Diagnostica, Taastrup, Denmark) directly on the plates. MIC values were measured by applying MIC test strip with amphotericin B, flucytosine, caspofungin, ketoconazole, posaconazole, itraconazole, and voriconazole with a concentration range of 0.002–32 *μ*g, and fluconazole with a concentration range of 0.016–256 *μ*g (Antifungal Discs, Liofilchem Diagnostici, Via Scozia, Italy). A single MIC test strip was applied per plate, while 4–6 discs were applied per plate and incubated for 3–7 days at 37°C.

### 2.4. DNA Extraction, PCR Amplification, and Sequencing

DNA was extracted using a standard cetyltrimethyl ammonium bromide (CTAB) procedure, as described previously [19]. The internal transcribed spacer (ITS) region with primers ITS1-F [20] and ITS4 [21] was amplified with Taq polymerase. Partial translation elongation factor (tef-1*α*) gene was amplified with primers 983F [22] and 2218R [23]. The D1/D2 domains of the large-subunit (28S) rRNA gene were amplified and sequenced using the primer pair ITS1/TW14 [21, 24]. All reactions were performed in an Eppendorf Gradient MasterCycler (Eppendorf, Hamburg). Conditions for amplification of ITS and D1/D2 domains: 95°C for 5 min; 35 cycles of 95°C for 30 s, 54°C for 30 s, and 72°C for 90 s; and finally 5 min at 72°C. Touchdown amplification of tef-1*α* was performed as follows: 95°C for 5 min; 9 cycles of 30 s at 95°C, 30 s at 66°C (−1°C every cycle), and 1 min at 72°C; followed by 30 cycles of 30 s at 95°C, 30 s at 56°C, and 1 min at 72°C; and a final elongation step of 7 min at 72°C.

The PCR products were sequenced with the same primers used for the PCR amplifications (Microsynth AG, Balgach, Switzerland). All sequences obtained in this study were deposited in GenBank. For information on fungal strains used in this study see Table 1. This table provides GenBank accession numbers to ITS, tef-1*α*, and D1/D2 domains of 28S rRNA gene (LSU) sequences for all accepted species in the genus *Saksenaea*.

### 2.5. Phylogenetic Analysis

For phylogenetic analysis, sequences were aligned with Clustal X [25]. Phylogenetic analysis was done with SeaView 4.6 [26] software. The phylogenetic tree was constructed using maximum likelihood (ML) method in SeaView and genetic distances were computed with the Kimura-2‐parameter (K2P) model. Bootstrap analyses were performed in ML with 1000 bootstrap replicates. *Apophysomyces elegans* ex-type strain CBS 476.78 was selected as outgroup for phylogenetic evaluation.

## 3. Results

### 3.1. Phylogenetic Analysis

Based on a search of NCBI's GenBank nucleotide database, the closest hits using the ITS sequence were *S. trapezispora* (UTHSC DI 15-1; Genbank: NR_147690; Identities = 601/644 (93%), gaps 23/644 (3%)) and *S. oblongispora* (CBS 133.90; Genbank: NR_137569; Identities = 595/652 (91%), gaps 35/652 (5%)). Using the tef-1*α* sequence, the closest hits were *S. oblongispora* (CBS 133.90; Genbank: HM776687; Identities = 473/476 (99%), no gaps) and *S. trapezispora* (UTHSC DI 15-1; Genbank: LT607408; Identities = 467/476 (98%), no gaps)). A region of the 28S rRNA containing D1 and D2 regions (LSU) showed highest similarities with *S. trapezispora* (GenBank NG_060019; Identities = 704/720 (98%), gaps 1/720 (0%)) and *S. oblongispora* (GenBank NG_057868; Identities = 702/719 (98%), no gaps).

**Figure 1:**
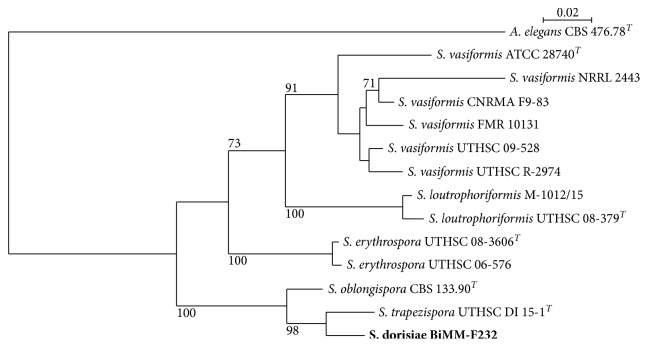
Maximum Likelihood tree based on a concatenated set of 3 sequences (ITS, LSU, and tef-1*α*) for the new taxon *S. dorisiae* is compared with species from the same genus *Saksenaea*. Numbers at nodes indicate bootstrap values (expressed as percentages of 1000 replications). *Apophysomyces elegans* CBS 476.78 was used as an outgroup. Scale bar indicates 0.02 substitutions per nucleotide position. *S* = *Saksenaea*, *A* = *Apophysomyces*; ^*T*^ex-type strain. New species is in bold.

The phylogenetic tree built by combining ITS, LSU, and tef-1*α* (Figure 1) indicates that the isolate represents a new species, being closest to *S. trapezispora*.

### 3.2. Taxonomy

**Figure 2:**
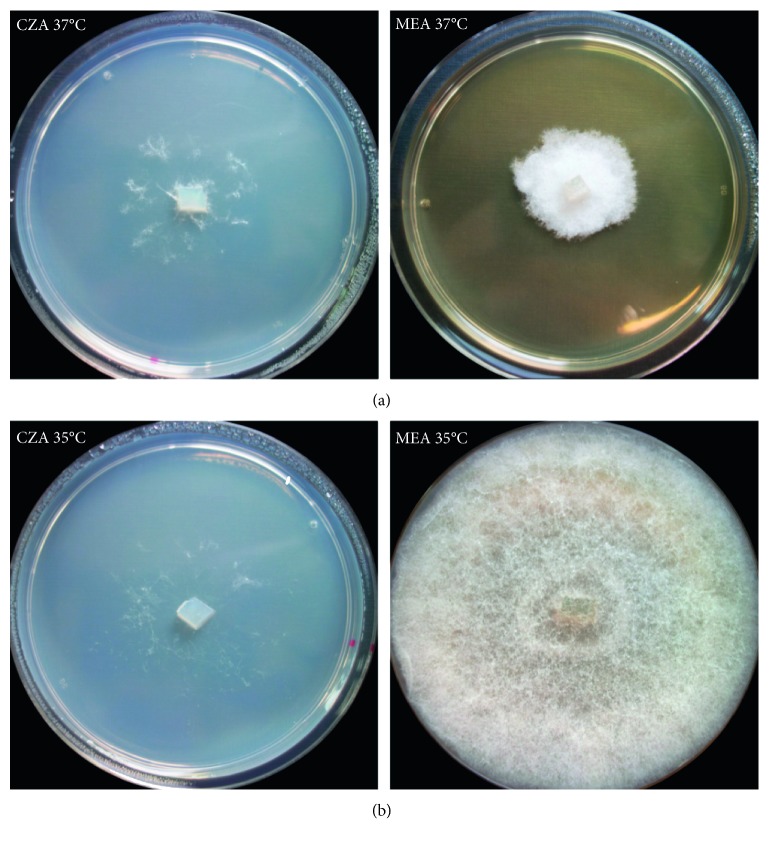
*Saksenaea dorisiae* (BiMM-F232). Colonies on CZA and MEA (4 days old) at 35 (a) and 37°C (b).

**Figure 3:**
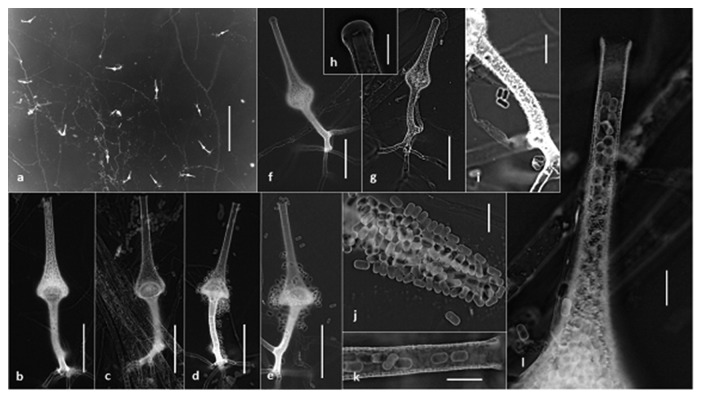
*Saksenaea dorisiae* (BiMM-F232). (a)–(e) Sporangiophores with sporangia (on CZA, 6 days old). (f)–(h) Young sporangia with rounded neck (closed). (i) Details of asperulate sporangiophore (on CZA, 6 days old). (j)–(l) Sporangiospores and details of sporangial neck (on CZA, 6 days old). Scale bars = 500 *μ*m (a), 50 *μ*m (b)–(g), 10 *μ*m (h)–(l).

**Figure 4:**
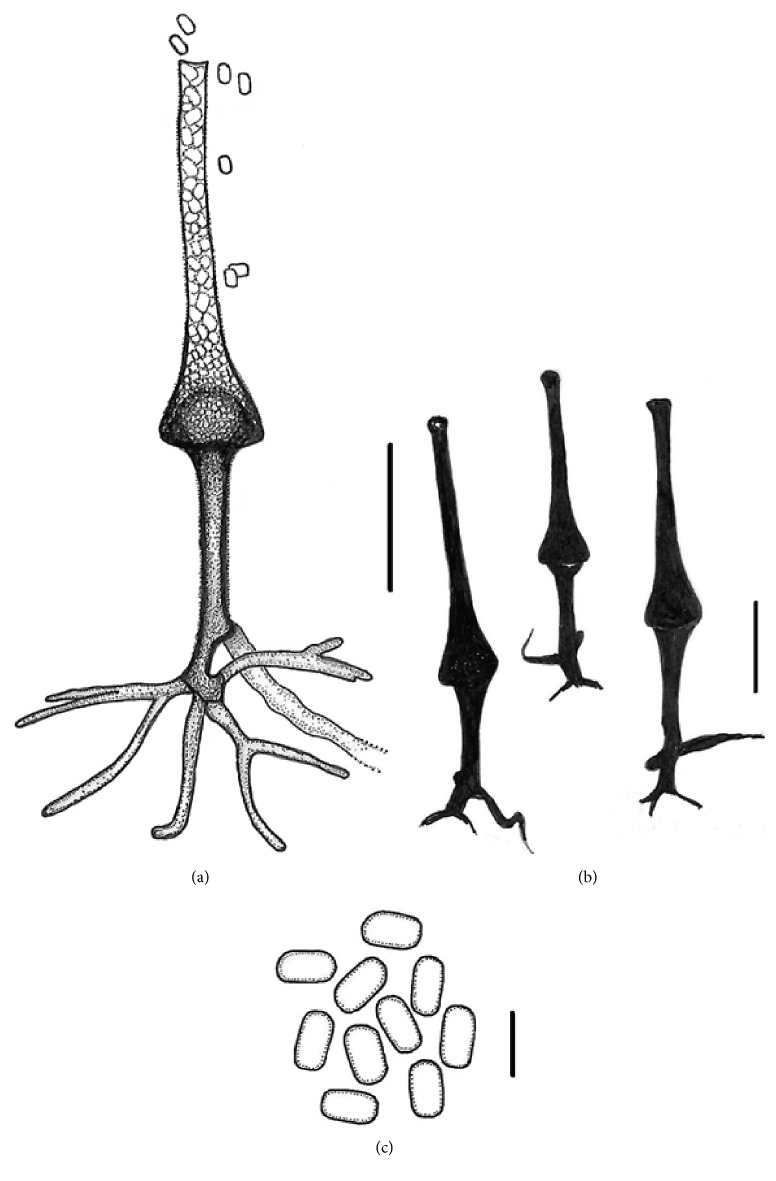
Line drawing of micromorphology of *Saksenaea dorisiae* (BiMM-F232). (a, b, and c) sporangiophores, sporangia, and sporangiospores on CZA (6–8 days old). (a) Sporangiophores with sporangium and mature sporangiospores. (b) Sporangiophores with sporangia (*in situ*). (c) Sporangiospores. (a, b) Scale bar = 50 *μ*m; (c) scale bar = 5 *μ*m.

 
*Saksenaea dorisiae* R. Labuda, A. Bernreiter, C. Schüller, J. Strauss & M. Wagner *sp. nov.* (Figures 2–4).
  MycoBank MB 830072
 
*Etymology*: Latin, *dorisiae* = named after Doris Hochenauer, who isolated the fungus.

### 3.3. Culture Characteristics (Figure 2)

Colonies reaching 25–30 mm in diameter after 4 d of incubation at 37°C on CZA, whitish, with very scarce (cobweb-like) aerial mycelium, with colorless reverse. Colonies at 37°C on MEA, CYA, PDA, CMA, and SDA more abundant floccose, mycelium moderately slow growing (30–65 mm) after 4 days, with colorless reverse, remaining sterile (without sporulation).

The optimum temperature for growth on CZA was between 20 and 35°C (45–60 mm diameter), reduced growth was observed at 15°C (15–20 mm diameter), and 37°C (25–30 mm diameter). Minimum growth was observed at 12°C (3–4 mm diameter), and the maximum temperature for growth was 39°C (0.5–1 mm diameter). The fungus did not grow at 40°C on any of the media used (CZA, CYA, MEA, and PDA). Overall growth on CYA, MEA, and PDA was approximately 20–25% faster after 4 days of incubation at the optimum temperatures compared to CZA ([Supplementary-material supplementary-material-1]). Germination and growing ability of the spores, mycelial pellets, and mycelium, after exposure at 5°C and 40°C is presented in Tables [Supplementary-material supplementary-material-1] and [Supplementary-material supplementary-material-1].

### 3.4. Micromorphology (Figures 3 and 4)

Hyphae mostly coenocytic (non-septate), branched, hyaline, smooth and thin walled, and up to 22 *μ*m wide. Sporangiophores erect, generally arising singly, unbranched, brown, (75-) 85–100 (-130) × (6-) 7–10 (-12) *μ*m, slightly to distinctively verrucose (asperulate) covered with bacilliform protuberances, terminating into hemispherical columellae (15–35 *μ*m wide), and with sparse, dichotomously branched rhizoids (root-like structure). Sporangia terminal, multispored, hyaline, flask-shaped (vasiform), slightly to distinctively verrucose (asperulate), (70-) 90–160 (-190) *μ*m long, at maturity with a conical venter up to 55 *μ*m wide (mostly 40–50 *μ*m), and gradually narrowing into a long neck (70–100 *μ*m × 6–10 *μ*m) with a rounded apex (closed with a mucilaginous plug) in young sporangia (Figures 3(f)–3(h)), truncated and opened at maturity. Sporangia observed after 5 days on CZA most abundantly at 30°C (up to 30–50 per plate), less so at 25 and 35°C (up to 10 per plate), while none at 20 and 37°C. The sporangia were formed at the center of the colony nearby or on agar block used for inoculation. Sporangiospores during development and at maturity (4–8 days) mainly short-cylindrical (capsulate) with rounded ends, a few also more or less trapezoidal in lateral view, smooth, thin walled, and hyaline, (4.5-) 5.0–5.5 (−6.0) × (2.0-) 2.5–3.0 (-3.5) *μ*m (mean = 5.1 ± 0.4 × 2.8 ± 0.3 *μ*m, *n* = 70). Zygospores were not observed.

The main distinguishing phenotypic characteristics of the new species compared with the other taxa of the genus *Saksenaea* are listed in the Table 2.

 
*Holotype*. The Republic of Serbia, Manastirica (Petrovac) isolated from a private, 65 m deep-well water sample (Code DOO33) 08. 10. 2018, isolated by Doris Hochenauer; PRM 951593 (Holotype, dried culture).
 
*Ex-Type Strain*. BiMM-F232 = CCF 6174.
 
*DNA Sequences*. GenBank MK559697 (ITS), GenBank MK569515 (tef-1*α*), and GenBank MK570305 (LSU).

### 3.5. *In Vitro* Antifungal Susceptibility Testing

**Figure 5:**
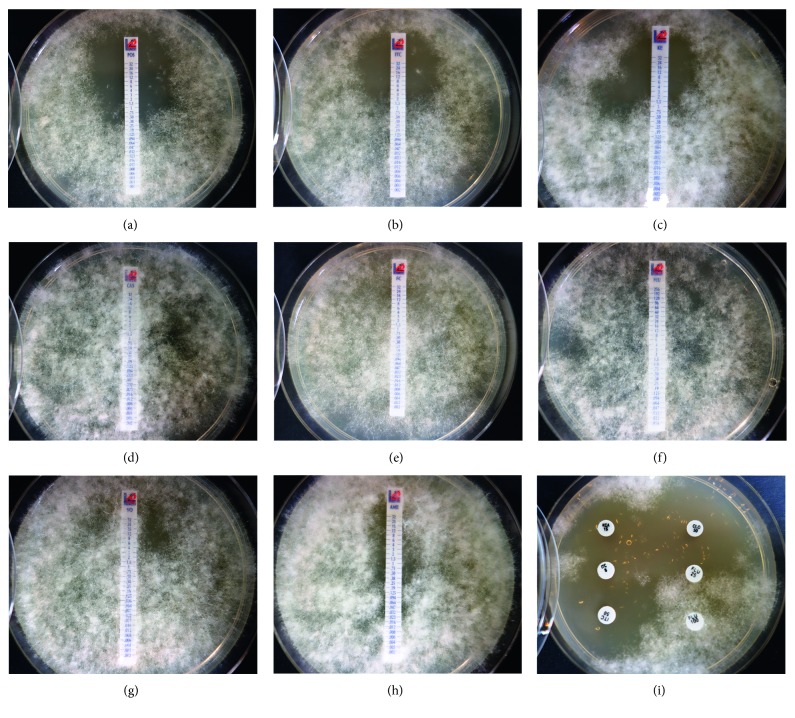
*In vitro* antifungal susceptibility of *Saksenaea dorisiae* BiMM-F232 towards selected antifungals after 3 days incubation at 37°C. MIC Test strips: (a) posaconazole (POS), (b) itraconazole (ITC), (c) ketoconazole (KE), (d) caspofungin (CAS), (e) flucytosine (FC),(f) fluconazole (FLU), (g) voriconazole (VO), (h) amphotericin B (AMB), and (i) antifungal discs(left top to left bottom: ketoconazole KCA 15 *μ*g and itraconazole ITC 8 and 15 *μ*g; right top to right bottom: clotrimazole CLO 50 *μ*m and fluconazole FLU 25 and 100 *μ*m); all plates were overgrown by the fungus after prolonged incubation (5 days) at 37°C.

The MIC test strips with eight antifungals used for the *Saksenaea* strain BiMM-F232 sensitivity testing showed that the fungus is sensitive towards ketoconazole, posaconazole, and itraconazole at rather high concentrations, i.e., 3.0, 2.0, and 4.0 *μ*g/mL, respectively. The fungus showed resistance towards caspofungin, flucytosine, and voriconazole (MIC >32 *μ*g/mL), as well as fluconazole (>256 *μ*g/mL). The MIC value for amphotericin B was unclear as no clear inhibition zone was formed (Figure 5). Additional application of 15 antifungals with defined concentrations per disc revealed sensitivity of the strain towards ciclopirox (25 mm zone at 50 *μ*g), clotrimazole (25 mm zone at 50 *μ*g), econazole (15 mm zone at 10 *μ*g), and terbinafine (25 mm zone at 30 *μ*g). The antibiotic discs containing amphotericin B (with 10 and 20 *μ*g), fluconazole (20 and 100 *μ*g), flucytosine (1 *μ*g), griseofulvin (10 *μ*g), miconazole (10 *μ*g), and voriconazole (1 *μ*g) showed limited (up to 8 mm) or no activity at all (Table 3). All antifungals strips and/or discs, except terbinafine and ciclopirox, were overgrown after incubation for 5 days (37°C). The same type of multiresistance towards all antifungals (except terbinafine) was also observed when mycelial pellets of the fungus were used for antifungal susceptibility test during this study.

## 4. Discussion

Phenotypically, the new species, *S. dorisiae*, differs from the other taxa in the genus *Saksenaea* S. B. Saksena (Mucorales, Saksenaeaceae) by the combination of the following features: (1) no growth at 40°C and slow to moderate growth at 15 and 37°C (15–20 mm and 25–30 mm, respectively), (2) conical sporangial venter, (3) morphology of sporangiospores (short-cylindrical, av. = 5.0 × 3.0 *μ*m), and (4) sparse rhizoids. The shape of sporangia and sporangiospores are very distinctive, rendering *S. dorisiae* to be easily discernable from the other hitherto known species of the genus. Phylogenetic analysis of the combined ITS, LSU, and tef-1*α* sequences showed the new species clustered with *S. oblongispora* and *S. trapezispora*, being closest to the later species (Figure 1). All three species do not grow at 42°C, in contrast to other *Saksenaea* species [1, 11]. At the time of this study, the genus *Saksenaea* contains 6 species, including the new taxon. Each of them is characteristic by a particular combination of morphological traits (mainly morphology of sporangia and sporangiospores). Thus, they are easily distinguished from *S. dorisiae* phenotypically. The conical-shaped sporangia, sparse rhizoids as well as capsulate sporangiospores formed by the new species are very distinctive, morphologically separating this fungus from all the others in the genus. Since the formation of sporangia is necessary for phenotypical identification of isolates into the genus and/or species level, incubation on CZA at 30°C are conditions conducive for sporulation. All species of *Saksenaea* are known for their rapid growth on traditional microbiological media (e.g., SAB, PDA, MEA, and WA) but without sporulation. To stimulate sporulation, Padhye and Ajello [18] suggested floating agar blocks in water with yeast extract, but we could not reproduce this method with *S. dorisiae*. Out of the 9 media used, only CZA was effective in stimulating sporangia development within 4–5 days, at 30°C. This medium has been used for morphological description of all known species of *Saksenaea* [1, 10, 11].

Compared to the phylogenetically close *S. trapezispora* [10], the new species grows substantially slower on CZA at 15°C and 37°C after 4 days (15–20 mm vs. 35–45 mm and 25–30 mm vs. 35–40 mm, respectively). In comparison to *S. dorisiae*, *S. trapezispora* has longer sporangiophores (up to 230 *μ*m vs. 130 *μ*m), spherical instead of conical sporangia, profuse rhizoids versus sparse ones, and substantially larger trapezoid sporangiospores (av. = 7 × 3.5 *μ*m) versus capsulate ones (av. = 5 × 3 *μ*m). In *S. trapezispora*, growth was similar to *S. dorisiae* on CZA at its optimal temperature (30–35°C) after 4 days of incubation. The other *Saksenaea* species grow very fast on this medium and their mycelium totally covers the 9 cm Petri dish after 4 days incubation at their optimal temperatures.

Apart from the growth characteristics, shape and size of sporangiospores are important distinguishing morphological characters within the genus [1, 10, 11]. Sporangiospores of *S. lautrophoriformis* and *S. vasiformis* species complex are long, cylindrical (to bacilliform), and up to 7 × 3.5 *μ*m large. In *S. erythrospora* they are biconcave (erythrocyte-like) up to 5.5 × 3 *μ*m large, in *S. oblongispora* ellipsoidal to oblong and up to 6.5 × 4.5 *μ*m large, while in *S. trapezispora* they are trapezoidal and up to 8 × 4 *μ*m large. The new species mostly form short cylindrical (capsulate) sporangiospores up to 6 × 3 *μ*m large.

The phylogenetically closest species, *S. oblongispora* [1], *S. trapezispora* [10], and *S. dorisiae*, are represented by only a single strain. *Saksenaea oblongispora* strain CBS 133.90 was isolated from a forest soil in Brazil, while *S. trapezispora* strain UTHSC DI 15-1 was isolated from the knee wound of a soldier in Texas, USA.

Mucormycoses caused by *Saksenaea* are difficult to treat. We analyzed sensitivity to antifungal drugs and found that *S. dorisiae* overgrew (with the exception of terbinafine and ciclopirox) all antifungals strips and discs used after prolonged incubation (5 days at 37°C). Of the 15 antifungals tested, terbinafine showed stable activity towards the mycelial form (growth from mycelial pellets) of the fungus even after prolonged incubation. It is interesting to note that amphotericin B, a drug that is being the first choice for treatment of mucormycoses [2], including those caused by the *Saksenaea* species (e.g. 6–9), showed very weak activity against *S. dorisiae*. From the azoles used in the study, itraconazole, ketoconazole, and posaconazole showed weak activity at rather high minimum effective concentrations, 3.0, 2.0, and 4.0 *μ*g/mL, respectively. Azoles, such as posaconazole, have been proposed as second-line agents against mucormycosis or as an alternative to amphotericin B in situations when high toxicity of amphotericin B has been detected [2]. As indicated here by *in vitro* tests, in case of potential infections caused by *S. dorisiae*, traditional antibiotic therapy of amphotericin B and posaconazole may not be the optimal treatment strategy. Similarly, in his review on *Saksenaea*, Dannaoui [13] stated that these fungi seem to be less susceptible to amphotericin B than other Zygomycetes. Noteworthy, we found that the fungus is not able to grow at 40°C and is rapidly inactivated at this temperature. After 1 day exposure at 40°C there was no growth or spore germination observed and further cultivation under optimal temperatures demonstrated that the fungus did not survive this heat treatment. This physiological limitation of the fungus could potentially be used as a subsidiary treatment strategy, along with the suggested terbinafine and/or ciclopirox therapy.

Even though *S. dorisiae* has been isolated from a single water sample of a private well located in a rural area of the Republic of Serbia (in the village of Manastirica), there is no clear evidence on its connection to water as a primary habitat. It is most likely that the fungus originated from the surrounding soil which polluted the ground water in the well. In fact, soil is supposed to be the primary habitat of *Saksenaea* and a known source for infection typically seen after skin rupture and subsequent contact with contaminated soil [3, 13, 27] or contaminated water [2, 4]. Although the presence of the *Saksenaea* fungi (as *S. vasiformis*) associated with soil environments has been rarely reported, the fungus has been found in different parts of the world, suggesting a wide distribution [13]. Species of this genus have been isolated from a clay loam soil in Tamil Nadu state (India), soil from forest tree nurseries in Georgia (the USA), soil in Barro Colorado Island (Panama), and in turtle nest sand on the Nancite beach (Costa Rica). Furthermore, they have been reported from soil of banana-producing areas in Cortes province (Honduras), soil of groundnut fields in Israel, soil sample in I-Lan prefecture (Taiwan), pineapple field soil in Okinawa (Japan), and also in Africa from intertidal driftwoods found in Mitsiua (Ethiopia). The infection associated with water or water environment of human and animals (dolphins and a killer whale) have also been reported previously [3, 4, 28].

The results of bacteriological investigation (unpublished data) of the water sample revealed a high level contamination by enterococci and other fecal bacterial species indicating that the water of this well was of poor microbiological quality not suitable for consumption. It is noteworthy that the fungus was isolated from an area characterized by cold winters typical for moderately continental climate. Interestingly, we found that the mycelium of the fungus and mycelial pellets (microcolonies in a liquid medium) did not survive at 5°C for more than 2 and 7 days, respectively. However, spores in a suspension in water were viable at low temperature for up to 50 days. Thus, especially during cold seasonal periods, spores are vital for propagation and survival of this fungus.

To date, all known *Saksenaea* species, were described from outside of Europe, namely, *S. vasiformis* (India: NRRL 2443), *S. lautrophoriformis* (USA: UTHSC 08-379 and India: M-1012/15), *S. erythrospora* (USA: UTHSC 08-3606 and Middle East: UTHSC 06-576), *S. oblongispora* (Brazil: CBS 133.90), and *S. trapezispora* (USA: UTHSC DI 15-1). To the best of our knowledge, reports dealing with *Saksenaea* fungi from Europe have been associated only with *S. vasiformis*. These reports on clinical cases are limited to the Mediterranean climate area of Spain [3, 14–16], Greece [2], and France [1, 13]. Hence, *S. dorisiae* represents the first described species of this genus originating from a moderately continental climate area in Europe. This is the first nonclinical report on the occurrence of the *Saksenaea* genus in European environment.

## 5. Conclusion

The study describes a novel species of the opportunistic pathogenic genus *Saksenaea* (*S. dorisiae*), isolated from an environmental sample (water from a private well) in the Republic of Serbia in October 2018. A polyphasic approach, which included multilocus (ITS, LSU, and tef-1*α*) analysis, revealed that the novel taxon is closely related to *S. trapezispora* and *S. oblongispora.* Despite its sensitivity to several conventional antifungals, the fungus can potentially evoke clinically challenging infections. *Saksenaea dorisiae* represents the first novel taxon of the genus described from Europe.

## Figures and Tables

**Table 1:** List of strains included in the study.

Strain^*a*^	Source	GenBank accession no.		
		ITS	Tef-1*α*	D1/D2 domains of 28S rRNA gene (LSU)
*Saksenaea dorisiae* BiMM-F232^*T*^	Water from a private well, the Republic of Serbia	**MK559697**	**MK569515**	**MK570305**
*Saksenaea vasiformis* FMR 10131	Cutaneous lesion, Tarragona, Spain	FR687326	HM776689	HM776678
*Saksenaea vasiformis* CNRMA F9-83	Skin lesions, France	FR687325	HM776688	HM776677
*Saksenaea vasiformis* NRRL 2443^*T*^	Soil, India	FR687327	HM776690	AF113483
*Saksenaea vasiformis* UTHSC 09-528	Human tissue, USA	FR687329	HM776692	HM776681
*Saksenaea vasiformis* UTHSC R-2974	Human tissue, USA	FR687332	HM776695	HM776684
*Saksenaea vasiformis* ATCC 28740	Craniofacial tissue and brain, USA	FR687322	HM776685	HM776674
*Saksenaea loutrophoriformis*M-1012/15	Palate necrotic tissue, India	LT796164	LT796166	LT796165
*Saksenaea loutrophoriformis* UTHSC 08-379^*T*^	Eye, USA	FR687330	HM776693	HM776682
*Saksenaea erythrospora* UTHSC 08-3606^*T*^	Bovine fetus, USA	NR 149333	HM776691	NG 059935
*Saksenaea erythrospora* UTHSC 06-576	Blood, Middle East	FR687331	HJN206536M776694	HM776683
*Saksenaea oblongispora* CBS 133.90^*T*^	Forest soil, Brazil	JN206283	HM776687	NG 057868
*Saksenaea trapezispora* UTHSC DI 15-1^*T*^	Knee wound, USA	NR 147690	LT607408	NG 060019
*Apophysomyces elegans* CBS 476.78^*T*^	Soil, India	NR 149336	AF157231	JN206536

^*a*^FMR, Facultat de Medicina in Ciències de la Salut, Reus, Spain; CNRMA, Centre National de Référence Mycologie et Antifongiques, Paris, France; NRRL, ARS culture collection, Peoria, IL, USA; UTHSC, Fungus testing laboratory, University of Texas Health Sciences Center, San Antonio, TX, USA; ATCC, American Type Culture Collection, Manassas, VA, USA; M-1012/15 = FMR 14516; CBS, Westerdijk Fungal Biodiversity Centre, Utrecht, the Netherlands; BiMM, Bioactive Microbial Metabolites unit, UFT-Tulln, Austria; ^*T*^ex-type strain. Newly obtained data are in bold.

**Table 2:** Comparison of the main phenotypic characteristics of *Saksenaea* spp.

Species	Growth at 37°C on CZA after 4 d (mm)	Growth at 42°C	Sporangiophore length (*μ*m)	Shape of mature sporangia-venters ^*∗∗*^	Spore size (*μ*m)	Spore shape	References
*S. dorisiae sp. nov.*	25–30	−	75–130	Conical	4.5–6 × 2.5–3	Short cylindrical -capsulate	This study
*S. erythrospora*	>85	+	100–150	Spherical	5–5.5 × 2.5–3	Ellipsoid-biconcave	[1]
*S. loutrophoriformis*	>85	+	50–75	Spherical	3.5–7 × 2–3.5	Long cylindrical	[11]
*S. oblongispora*	>85	−	80–100	Spherical	5–6.5 × 3–4.5	Oblong	[1]
*S. trapezispora*	35–40	−	150–230	Spherical	5.5–8 × 3.5–4	Trapezoid	[10]
*S. vasiformis* complex	>85^*∗*^	+	65–100	Spherical	5–7 × 2–3	Long cylindrical	[1]

^*∗*^Filling the Petri dish (diameter, 9 cm); ^*∗∗*^shape of sporangia listed are based on available drawings and illustrations in the referred studies.

**Table 3:** *In vitro* antifungal susceptibility of *Saksenaea dorisiae* (BiMM-F232) towards 15 antifungal discs after 3 days of incubation at 37°C.

Antimycotic	Antifungal disc (concentration *μ*g/disc)	Spores resistant/sensitive (mm zone)	Mycelial pellets resistant/sensitive (mm zone)
Amphotericin B	20	R/S (9)^*∗*^	R
Caspofungin	5	R	R
Ciclopirox	50	S (25)	R
Flucytosine	1	R	R
Griseofulvin	10	R	R
Nystatin	100 IU	R/S (6)^*∗*^	R
Terbinafine	30	S (25)	S (30)
Clotrimazole	50	S (25)^*∗*^	R
Econazole	10	S (15)^*∗*^	R
Fluconazole	100	R	R
Itraconazole	8	S (15)^*∗*^	R
Ketoconazole	15	R/S (8)^*∗*^	R
Miconazole	10	R/S (4)^*∗*^	R
Posaconazole	5	S (15)^*∗*^	R
Voriconazole	1	R	R

R = -resistant; S = sensitive. Responses with an asterisk^*∗*^ indicate re-growing of the fungus into the inhibition zones after prolonged incubation (5 days at 37°C); spores (4.0 × 10^5^ CFU/mL) and mycelial pellets in size of 50–200 *μ*m in diameter (2.0 × 10^4^/mL).

## Data Availability

The sequence data used to support the findings of this study have been deposited in the NCBI Genbank repository (https://www.ncbi.nlm.nih.gov/genbank/). The description of new species (registration) data used to support the findings of this study have been deposited in the Mycobank repository (http://mycobank.org/).

## Supplementary Materials

Supplementary Materials Supplementary Table S1: effect of low temperature (5 ± 0.2°C) on viability of *Saksenaea dorisiae* (BiMM-F232). Table S2: effect of high temperature (40 ± 0.1°C) on viability of *Saksenaea dorisiae* (BiMM-F232). Figure S1: temperature dependent growth of *Saksenaea dorisiae* (BiMM-F232).
