# Supplementary material for: Saksenaea dorisiae sp. nov., a New Opportunistic Pathogenic Fungus from Europe
Source: Int J Microbiol. 2019 Sep 22;2019:6253829. doi: 10.1155/2019/6253829 (PMC6778886; doi:10.1155/2019/6253829)
Supplement: Supplementary Materials — Supplementary Table S1: effect of low temperature (5 ± 0.2°C) on viability of Saksenaea dorisiae (BiMM-F232). Table S2: effect of high temperature (40 ± 0.1°C) on viability of Saksenaea dorisiae (BiMM-F232). Figure S1: temperature dependent growth of Saksenaea dorisiae (BiMM-F232). [file 6253829.f1.pdf]

SUPPLEMENTAL INFORMATION

Table S1: Effect of low-temperature (5±0.2°C) on viability of *Saksenaea dorisi*ae (BiMM-F232)

Fungus	1 d	2 d	3 d	4d	7 d	14d	21d	28d	42d	50d
Spores*	+++	+++	+++	+++	+++	+++	+++	+++	+++	+++
Pellets **	+++	+++	+++	+++	+	-	-			
Mycellium***	+++	++	-	-						

Growth: +++ = maximum (very good); ++ = good; + = poor; - = none; * spores (4.0 x 10^5 CFU/mL physiologic solution) collected from CZA; 100 µL applied on plate (MEA, 37°C); **micro-pellets of ca 50-200 µm in diam. ~ 2.0 x 10^4/mL) after 10-12 d incubation submerse YES5%, 140 rpm, 25°C; 100 µL applied on plate (MEA, 37°C); ***mycelium without sporulation; colony growing on MEA plate after 4 d incubation (37°C) exposed to low temperature 5±0.5°C °C

Table S2: Effect of high-temperature (40 ±0.1°C) on viability of *Saksenaea dorisiae* (BiMM-F232)

Fungus	40°C (24h)	40°C (48h)
Spores*	-	-
Pellets**	-	-
Mycelium***	+	-

Growth: + = poor; - = none; * spores (4.0 x 10^5 CFU/mL physiologic solution) collected from CZA (noticed in material and method); 100 µL applied on plate (MEA, 37°C); **micro-pellets of ca 50-200 µm in diam. ~ 2.0 x 10^4/mL) after 10-12 d incubation submerse YES5%, 140 rpm, 25°C; 100 µL applied on plate (MEA, 37°C); ***mycelium without sporulation; colony growing on MEA plate after 4 d incubation (37°C) exposed to high temperature (40 ±0.5°C)

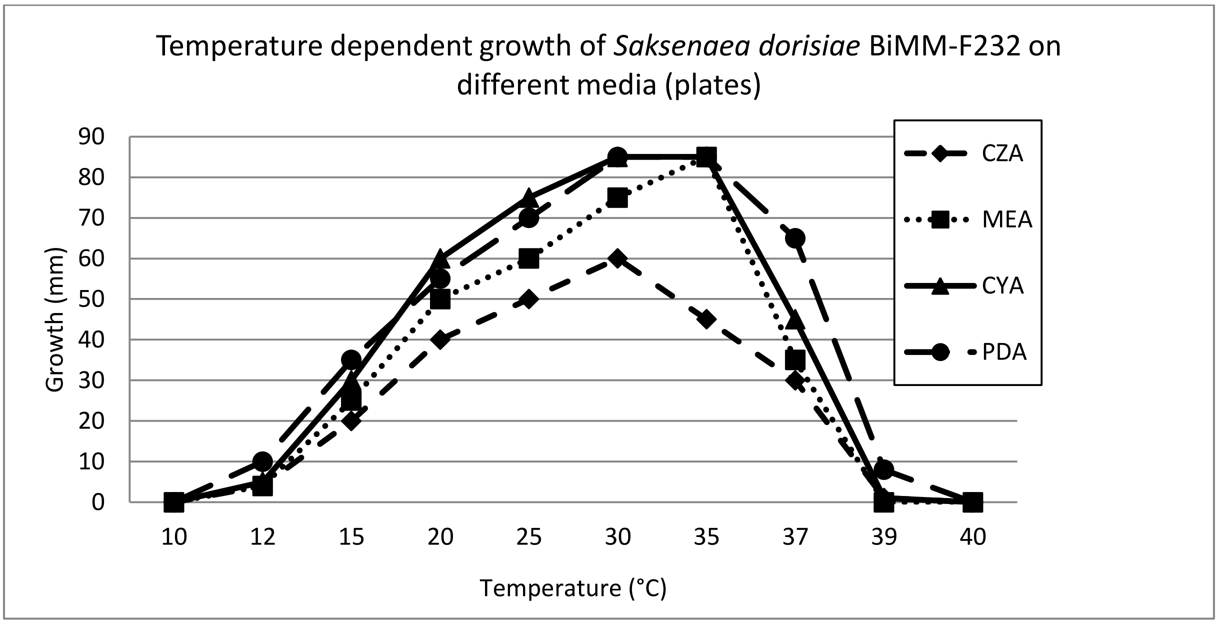

FIGURE S1: Temperature dependent growth of *Saksenaea dorisiae* (BiMM-F232) and its maximal colony extension (in mm) on Czapek agar (CZA), Malt extract agar (MEA), Czapek- yeast extract agar (CYA) and Potato dextrose agar (PDA)
